# The Two-Step Test as a Practical Discriminator of Independent Ambulation One Week After Total Hip Arthroplasty

**DOI:** 10.7759/cureus.92076

**Published:** 2025-09-11

**Authors:** Naoki Higashijima, Masanori Fujii, Saki Matsumoto, Yasuo Takei, Yosuke Oba, Masaya Ueno, Shunsuke Kawano

**Affiliations:** 1 Advanced Comprehensive Functional Recovery Center, Saga University Hospital, Saga, JPN; 2 Orthopaedic Surgery, Faculty of Medicine, Saga University, Saga, JPN; 3 Research Center of Arthroplasty, Faculty of Medicine, Saga University, Saga, JPN

**Keywords:** ambulation, assistive device, physical function, rehabilitation, total hip arthroplasty, two-step test

## Abstract

Background

This study aimed to identify physical function factors associated with independent ambulation without assistive devices one week after total hip arthroplasty (THA) in female patients.

Methodology

This retrospective cohort study included 162 female patients who underwent unilateral THA via a posterior approach for hip osteoarthritis between January 2020 and February 2023. Patients were classified into the following two groups based on the locomotion item of the Functional Independence Measure: the independent ambulation group (n = 92) and the dependent ambulation group (n = 70). Physical function was assessed one week postoperatively using knee extension strength, hip abduction strength, the 30-second chair stand test, the two-step test, and one-leg stance time.

Results

The independent ambulation group showed significantly better performance in all physical function measures, except knee extension strength on the operated side. Multivariate logistic regression identified the two-step value as the only independent factor associated with independent ambulation (odds ratio = 0.02; 95% confidence interval = 0.00-0.10; p < 0.001). The optimal cut-off value was 0.331, with an area under the curve of 0.87, a sensitivity of 80.0%, and a specificity of 93.5%.

Conclusions

The two-step test is a simple and clinically useful tool for identifying patients capable of independent ambulation without assistive devices in the early postoperative period after THA. A two-step value of 0.331 may serve as an objective indicator to support early rehabilitation planning. Future longitudinal studies are warranted to evaluate the relationship between early two-step test performance and long-term functional recovery after THA.

## Introduction

Osteoarthritis is a globally prevalent condition, affecting approximately 7.6% of the world’s population in 2020, with the burden expected to rise further due to an aging population [1]. Among musculoskeletal disorders, hip osteoarthritis affects nearly 20% of individuals over 50 years of age, with 4.2% experiencing symptoms such as pain, limited range of motion, and gait disturbance [2]. Total hip arthroplasty (THA) is an established surgical intervention for end-stage hip osteoarthritis, with favorable long-term outcomes [3].

Gait ability in the immediate postoperative period is one of the key determinants of readiness for hospital discharge directly to home after THA, alongside factors such as advanced age, obesity, and the availability of social support [4]. Furthermore, early postoperative gait performance has been associated with longer-term outcomes, such as the Forgotten Joint Score-12 at two years postoperatively [5]. Therefore, timely and effective rehabilitation aimed at restoring safe ambulation is a primary goal, particularly during the first week after THA.

Most patients undergoing THA rely on assistive devices during the early recovery phase to ensure safe mobility. On average, cane-assisted walking is initiated approximately 12.5 ± 5.0 days postoperatively [6], and up to 97% of patients undergoing THA via the posterior approach still require assistive devices two weeks after discharge [7]. While assistive devices, when prescribed appropriately, can enhance safe mobility, activity levels, and social participation, prolonged or unnecessary use can hinder recovery and contribute to an increased risk of falls or muscle disuse [8].

Previous studies on gait and assistive device use after THA have primarily evaluated ambulation as a binary marker of independence. However, ambulation status is influenced not only by physical function but also by environmental factors, psychosocial factors, cognitive factors, economic factors, and personal preference. Therefore, a comprehensive postoperative rehabilitation assessment that considers these elements is essential, and the establishment of objective, simple, and modifiable physical performance measures may provide additional value in determining the need for assistive devices. Such indicators could also support the development of more individualized physical therapy strategies to promote gait independence.

Therefore, the purpose of this study was to identify physical function factors associated with the need for assistive devices in female patients capable of unassisted ambulation one week after THA.

## Materials and methods

Study design

This study employed a retrospective cohort design. This study was conducted in accordance with the Declaration of Helsinki and relevant national guidelines for clinical research ethics. Ethical approval for this study was obtained from the Institutional Ethical Review Board of the Faculty of Medicine, Saga University (approval number: 2025-02-R-02; approval date: April 28, 2025), and the requirement for informed consent of the participants was waived by the Institutional Ethical Review Board of the Faculty of Medicine, Saga University, due to the retrospective nature of the study.

Patients

**Figure 1:**
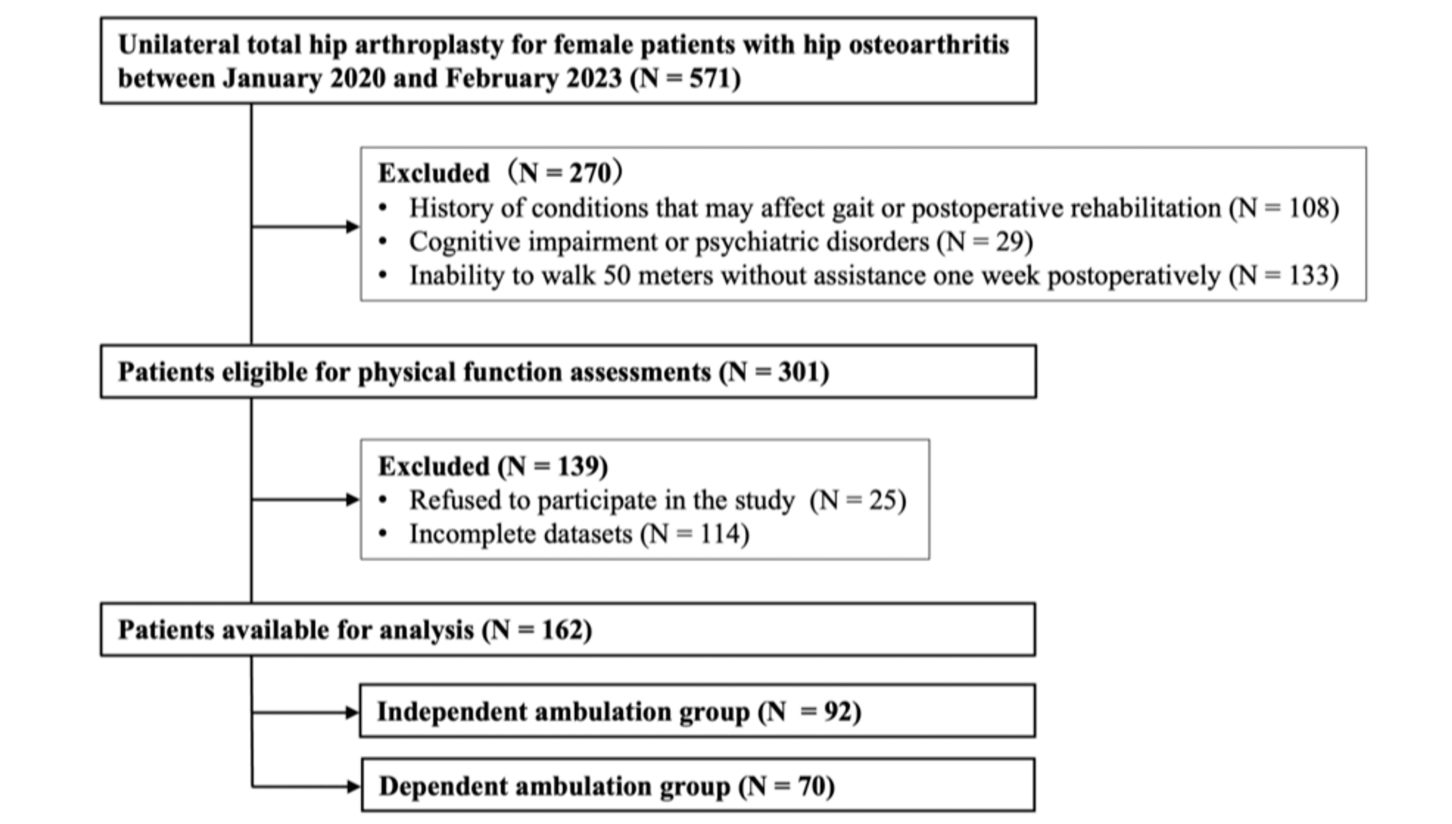
STROBE flow diagram of patient selection for this study. STROBE: Strengthening the Reporting of Observational Studies in Epidemiology

A total of 767 patients underwent primary THA via a posterior approach at our institution between January 2020 and February 2023. Of these, 571 female patients who underwent unilateral THA for hip osteoarthritis were included in this study. Exclusion criteria were a history of medical or orthopedic conditions affecting gait or postoperative rehabilitation (n = 108), cognitive impairment or psychiatric illness (n = 29), and inability to ambulate 50 m continuously without assistance one week postoperatively (n = 133). After applying these criteria, 301 patients were considered eligible for this study. An additional 25 patients who declined to participate and 114 with incomplete datasets were excluded. Therefore, a total of 162 female patients (mean age = 68.0 ± 9.0 years) were included in the final analysis (Figure 1).

Ambulation status was assessed using the locomotion (walking) item of the Functional Independence Measure (FIM) [9]. Patients who scored ≥6 (independent walking for ≥50 m, with or without assistive devices) were considered ambulatory. Those who scored 7 (completely independent, without assistive devices) were assigned to the independent ambulation group, whereas those who scored 6 (independent with assistive devices) were assigned to the dependent ambulation group.

Data collection and measurements

Baseline characteristics were collected by medical record review, including age, height, weight, body mass index (BMI), diagnosis, and radiographic stage of osteoarthritis according to the Japanese Orthopaedic Association (JOA) classification [10]. In addition, hip function was assessed using the JOA hip score, which consists of four subscales, i.e., pain (0-40 points), range of motion (0-20), gait (0-20), and activities of daily living (0-20), with a maximum total score of 100 points [11]. Higher scores indicated better hip function. The JOA hip score has been reported to correlate strongly with the Harris hip score [12].

Knee Extension Strength and Hip Abduction Strength

Isometric strength was measured bilaterally using a handheld dynamometer (Mobie MT-100/150; Sakai Med Co., Ltd., Tokyo, Japan) with a fixation strap method according to protocols with established reliability [13]. For knee extension strength, patients were seated with the knee flexed at 90°, and the sensor pad was placed on the distal anterior tibia. For hip abduction strength, patients were supine with the hip in neutral abduction/adduction, and the sensor was placed on the distal lateral thigh and stabilized with a strap around the contralateral thigh. The contralateral limb was manually supported by the examiner. For both tests, two five-second maximal isometric contractions were performed with at least 30 seconds of rest. The higher value was recorded and normalized to body weight (%BW) [14].

30-Second Chair Stand Test (CS-30)

Using a 40 cm high chair, participants started in a seated position with arms crossed over the chest and were instructed to stand up to full knee extension and sit back down as many times as possible within 30 seconds. A full cycle was defined as a complete stand and return to the seated position. The total number of cycles completed was recorded [15].

Two-Step Test

Participants took two maximal steps forward from a standing start while maintaining balance, stopping with both feet together at the end of the second step. The total distance was measured in centimeters and divided by the participant’s height to calculate the two-step value, an index that reflects stride length and dynamic balance [16].

One-Leg Stance Time

The time was measured on the operated side with participants standing, hands on their hips, and the non-operated leg raised approximately 5 cm. The time was recorded until the raised leg touched the floor or a significant loss of balance occurred. Two trials were performed ≥30 seconds apart, and the longer duration was recorded [17].

All scores and all physical function measures used in this study are publicly available or have been previously published with no restriction for academic use.

Postoperative rehabilitation protocol

All patients followed a standardized clinical pathway. Full weight-bearing was allowed on the first postoperative day. Physical therapy included hip range of motion exercises, muscle strengthening, gait training, and basic mobility training for up to 40 minutes daily. Patients were also instructed in self-directed exercise using printed brochures and instructional videos, and were educated on dislocation precautions. Assistive devices were selected by licensed physical therapists based on individual gait performance and safety considerations. All rehabilitation and assessments were supervised by physical therapists with more than 10 years of clinical experience.

Statistical analysis

Sample size was estimated using G*Power software [18]. Based on a two-group independent t-test with an effect size of 0.5, alpha of 0.05, and power of 0.80, the required sample size was calculated to be 128. All statistical analyses were performed using EZR (Saitama Medical Center, Jichi Medical University), a graphical interface for R specifically designed for biostatistics [19]. A two-tailed p-value <0.05 was considered statistically significant. Normality of distribution was assessed using the Shapiro-Wilk test. Categorical variables were compared using the chi-square test, and continuous variables were analyzed using either the independent t-test or Mann-Whitney U test, based on data distribution. Multivariate logistic regression was used to identify independent factors associated with the ability to ambulate without assistive devices. Variance inflation factors (VIFs) were calculated to assess multicollinearity; variables with VIF ≥5 were excluded. Receiver operating characteristic (ROC) curve analysis was performed for variables identified as significant, and optimal cut-off values were determined using the Youden index. The area under the curve (AUC) was used to assess model discrimination.

## Results

Baseline characteristics

Based on the FIM locomotion scores, 92 patients were assigned to the independent ambulation group and 70 to the dependent ambulation group (Figure 1). Table 1 summarizes the baseline characteristics of the two groups. The independent ambulation group was significantly younger than the dependent ambulation group (67.1 ± 7.9 vs. 70.2 ± 9.8 years, p < 0.001). There were no significant differences between the groups in height, body weight, BMI, or JOA classification for the operated or non-operated side, or in preoperative JOA hip scores.

**Table 1:** Comparison of baseline characteristics between the independent and dependent ambulation groups. Data are presented as mean ± SD, median (interquartile range) or number. *: JOA classification for hip osteoarthritis: 0, almost normal; 1, prearthrosis; 2, early stage; 3, advanced stage; 4, end stage. JOA: Japanese Orthopaedic Association; THA: total hip arthroplasty

Variables	All patients (N = 162)	Independent ambulation group (N = 92)	Dependent ambulation group (N = 70)	p value	t/χ²/U value
Age (years)	68.0 ± 9.0	67.1 ± 7.9	70.2 ± 9.8	0.026	t = 2.248
Height (cm)	152.0 (148.0-157.0)	153.0 (149.0-157.3)	152.0 (147.0-156.0)	0.241	U = 3776
Body weight (kg)	54.0 (47.0-60.0)	53.5 (47.8-59.3)	55.0 (46.0-60.8)	0.241	U = 3209
Body mass index (kg/m^2^)	23.0 (20.0-26.0)	22.5 (20.8-25.4)	22.9 (20.4-26.8)	0.172	U = 3035
JOA classification* (0/1/2/3/4/THA) operated side	0/0/5/38/119/0	0/0/3/25/64/0	0/0/3/23/39/0	0.940	χ² = 1.850
JOA classification* (0/1/2/3/4/THA) non-operated side	24/26/27/30/26/29	11/16/16/18/16/15	13/10/11/12/10/14	0.808	χ² = 2.148
JOA hip score	50.0 (40.0-61.0)	50.5 (40.8-61.3)	48.0 (37.3-57.0)	0.617	U = 3703

Physical function measures

Table 2 presents a comparison of physical function measures between the two groups. The independent ambulation group demonstrated significantly higher performance in knee extension strength on the non-operated side (p < 0.001), hip abductor strength on both the operated and non-operated sides (p < 0.001), CS-30 repetitions (p < 0.001), two-step value (p < 0.001), and one-leg stance time on the operated side (p = 0.008). No significant difference was found for knee extension strength on the operated side (p = 0.184).

**Table 2:** Comparison of physical function measures between the independent and dependent ambulation groups. Data are presented as mean ± SD or median (IQR).

Variables	All patients (N = 162)	Independent ambulation group (N = 92)	Dependent ambulation group (N = 70)	P-value	t/U value
Knee extension strength (operated side) (%)	24.9 (22.8-26.5)	24.9 (23.4-25.9)	24.8 (23.8-25.8)	0.715	U = 2915
Knee extension strength (non-operated side) (%)	29.4 ± 8.6	31.0 ± 7.7	27.4 ± 9.4	<0.001	t = 2.722
Hip abductor strength (operated side) (%)	10.2 (7.0-16.4)	12.5 (9.0-18.7)	7.8 (5.2-11.0)	<0.001	U = 4648
Hip abductor strength (non-operated side) (%)	14.8 (10.1-19.8)	16.1 (12.1-21.7)	12.6 (8.6-17.6)	<0.001	U = 4174
30-second chair stand test (times)	8.0 (0-11.0)	10.0 (7.0-12.0)	4.0 (0-9.0)	<0.001	U = 4733
Two-step value (cm/height)	0.7 (0-0.8)	0.7 (0.65-0.9)	0 (0-0)	<0.001	U = 5616
One-leg stance time (Operated side) (second)	2.0 (0-8.75)	5.0 (2.0-14.25)	0 (0-0)	0.008	U = 5381

Logistic regression analysis

Multivariate logistic regression analysis was performed with variables that showed significant differences in the univariate analysis. The two-step value was the only independent factor significantly associated with independent ambulation without assistive devices (odds ratio = 0.02, 95% confidence interval = 0.00-0.10, p < 0.001) (Table 3). No multicollinearity was found among the variables (all VIFs <5).

**Table 3:** Multivariate logistic regression analysis of factors associated with independent ambulation.

Variables	Odds ratio	95% confidence interval	P-value
Age	1.03	0.97-1.08	0.330
Knee extension strength (non-operated side)	0.99	0.93-1.05	0.700
Hip abductor strength (operated side)	0.89	0.78-1.03	0.058
Hip abductor strength (non-operated side)	1.05	0.94-1.18	0.350
30-second chair stand test	1.00	0.90-1.11	0.960
Two-step value	0.02	0.00-0.10	<0.001
One-leg stance time (operated side)	0.95	0.88- 1.03	0.220

ROC curve analysis

**Figure 2:**
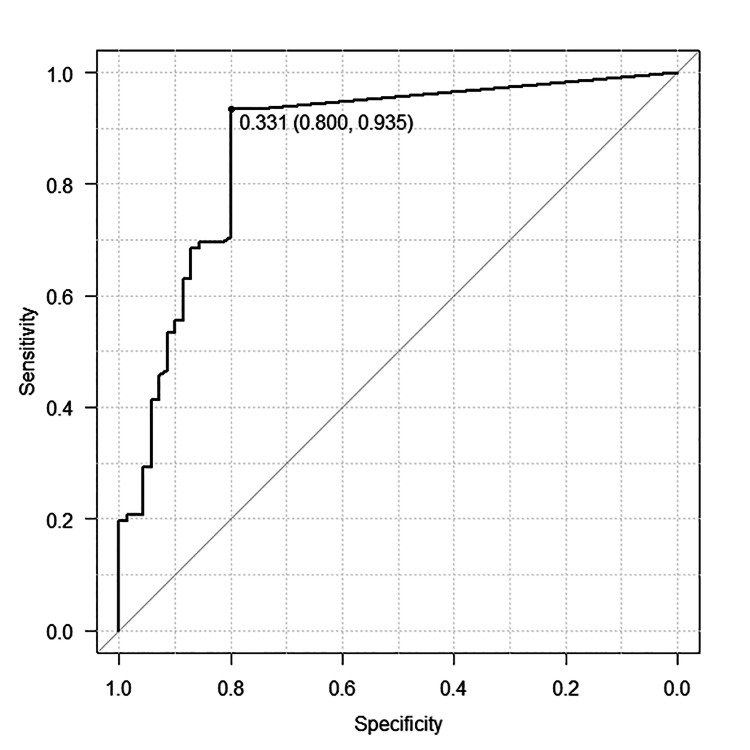
Receiver operating characteristic curve for the two-step value in discriminating independent ambulation without assistive devices. The optimal cut-off value was 0.331, with an area under the curve of 0.87, a sensitivity of 80.0%, and a specificity of 93.5%.

The ROC curve analysis for the two-step value yielded an AUC of 0.87, indicating excellent discriminatory ability. The optimal cut-off value determined using the Youden index was 0.331, with a sensitivity of 80.0% and a specificity of 93.5% (Figure 2).

## Discussion

This study examined the physical function factors associated with the need for assistive devices among female patients who could ambulate without personal assistance one week after THA. The key finding was that among several physical performance measures, including muscle strength, balance, and functional mobility, the two-step test was the only independent factor significantly associated with unassisted ambulation. Patients in the independent ambulation group demonstrated superior performance across several physical domains, and a two-step cut-off value of 0.331 was identified as a clinically relevant threshold, showing high sensitivity (80.0%) and specificity (93.5%), in discriminating between assistive device users and non-users. Although ambulation status and two-step performance were assessed simultaneously, these results suggest that the two-step test may serve as a practical and objective tool to complement functional assessments in the early stage of postoperative rehabilitation.

Patients in the dependent ambulation group were significantly older. This finding is consistent with previous studies identifying age as a key factor associated with assistive device use in community-dwelling older adults and postoperative THA populations [20,21]. Muscle strength, particularly knee extensor strength on the operative side, typically declines after THA and requires time to recover [22,23]. Weakness in this muscle group contributes to impaired gait [24] and has been recognized as a determinant of ambulation ability in frail elderly and cognitively impaired populations [25,26]. While the non-operative limb often retains greater preoperative strength, hip abductor weakness on the operative side is common and contributes to gait asymmetry and Trendelenburg gait [27-29]. Previous studies have also associated reduced abductor strength with a higher likelihood of using an assistive device [30].

The CS-30 test, a validated indicator of lower-limb muscle strength, is closely associated with sarcopenia and functional mobility in older adults [15]. Both sarcopenia and muscle weakness in the lower limbs are recognized risk factors for falls [31,32]. The two-step test, a composite measure of walking ability, balance, and lower extremity function, has been associated with walking ability, fall risk, and the use of assistive devices in elderly populations [33-35]. Similarly, one-leg stance time is a commonly used indicator of static balance and fall risk, with shorter stance times associated with impaired ambulation in frail older adults [26,36]. Thus, the significant differences observed in these measures between the two groups in our cohort are consistent with the existing literature and highlight the multifactorial nature of early postoperative ambulation ability.

Of all the examined variables, the two-step value emerged as the only independent factor associated with independent ambulation. This test reflects multiple physical domains, including muscle strength, flexibility, and balance, all of which are essential for safe, unassisted ambulation [37]. Previous studies have demonstrated an association between the two-step value and gait parameters, such as stride length, joint moment, and sagittal plane kinematics [38], as well as other functional mobility assessments, including the Timed Up and Go test and fall risk indices [34,37]. Because the two-step test is influenced by stride length, a well-known fall risk factor, it may help identify patients with limited hip extension or compromised gait stability who are more likely to rely on assistive devices [39,40].

The two-step cut-off value of 0.331 identified in this study is notably lower than that reported in community-based studies (e.g., 0.93) [35]. This discrepancy is likely due to the early postoperative time point of assessment, during which patients have not yet regained joint mobility or normalized gait [41]. Additionally, the FIM scoring system used to define independence in this study may reflect a more conservative threshold for ambulation than those applied in community mobility research. These findings suggest that the two-step test may serve as an objective and clinically applicable indicator for determining the need for assistive devices in the early postoperative phase after THA. A cut-off value of 0.331 may inform clinical decision-making regarding the timing of assistive device withdrawal and assist in the design of individualized rehabilitation strategies to promote independent ambulation.

Although the two-step test does not predict future ambulation status, it can serve as an efficient screening tool to help rehabilitation professionals tailor assistive device use based on physical function at a critical point in recovery. Its simplicity, minimal equipment requirements, and strong association with ambulation status make it especially suitable for busy inpatient rehabilitation environments.

Limitations

Several limitations should be acknowledged. First, this study was conducted at a single institution and included only patients undergoing posterior approach THA, which may limit generalizability. Second, the analysis was limited to female patients; therefore, the findings may not be applicable to male populations. Third, the specific types of assistive devices used were not recorded, and it remains unclear whether device selection was optimized in all cases. Finally, ambulation status and physical function were measured concurrently at one week, which precluded conclusions about predictive relationships. Future studies should examine these associations longitudinally, include diverse patient populations and surgical approaches, and evaluate the type and appropriateness of assistive devices.

## Conclusions

This study demonstrated that among female patients, one week after THA, the two-step test was significantly associated with the need for assistive devices. A two-step cut-off value of 0.331 demonstrated excellent discriminatory ability for identifying patients who could ambulate independently. While not predictive, the two-step test may serve as a simple, objective tool to support early rehabilitation planning and guide clinical decision-making regarding the need for assistive devices. Further longitudinal studies are warranted to assess how early two-step performance relates to longer-term functional recovery trajectories after THA.
